# Olaparib alleviates diclofenac-induced toxicity in HepG2 cells via modulation of oxidative stress and mitochondrial functions

**DOI:** 10.1007/s11010-026-05581-3

**Published:** 2026-06-02

**Authors:** Mohamed Maki, Viola B. Vantus, Balazs Koszegi, Aliz Szabo, Katalin Fekete, Omeralfaroug Ali, Maysaa A. Ali, Tibor Z. Janosi, Ferenc Gallyas

**Affiliations:** 1 Department of Biochemistry and Medical Chemistry, Medical School, University of Pécs, Pécs, Hungary; 2 Department of Animal Physiology and Health, Institute of Physiology, and Animal Nutrition, Hungarian University of Agriculture and Life Sciences, Kaposvár, Hungary; 3 Division of Biotechnology, Department of Applied Sciences, University of Technology, Baghdad, Iraq; 4 Istvan Abraham Nano-Bio-Imaging Core Facility, Medical School, University of Pécs, Pécs, Hungary

**Keywords:** PARP, ROS, Oxidative phosphorylation, Mitochondrial membrane potential, Rotenone, NAC

## Abstract

Non-steroidal anti-inflammatory agents are widely used for their analgesic, anti-inflammatory, and antipyretic effects, and a prominent representative of the drug family, diclofenac has recently been proposed for cancer therapy. However, overdose of diclofenac has also been reported to cause liver toxicity, whereas olaparib, a widely used antineoplastic poly (ADP-ribose) polymerase inhibitor, is known for its mitochondria-mediated cytoprotective properties. In this study, we used HepG2 human hepatocellular carcinoma cells, which retain various characteristics of hepatocytes, to study the interaction between these two drugs. We found that diclofenac caused a 20–50% decrease in the viability and invasive growth of the cells, which olaparib ameliorated by approximately 50%. Additionally, olaparib reversed diclofenac-induced mitochondrial depolarization as demonstrated by JC-1 fluorescence microscopy and flow cytometry. Furthermore, although not possessing significant free-radical scavenging properties according to the cell-free Fenton reaction system, olaparib also mitigated diclofenac-induced reactive oxygen species production and apoptosis-inducing factor-mediated parthanatos. Using the Seahorse cellular respirometer, we showed that diclofenac shifted the energy production of HepG2 cells toward oxidative phosphorylation by approximately 40%, whereas olaparib reversed this effect, suggesting that the two drugs may have opposite effects on the metabolic reprogramming of cancer cells. Therefore, further studies are needed before considering their combined use for oncological applications. Additionally, we demonstrated that complex I inhibition and preservation of cellular NAD^+^ pool are unlikely to be involved among the mechanisms of olaparib’s protective effect.

## Introduction

Diclofenac, a well-known nonsteroidal anti-inflammatory agent, is recognized for its analgesic, anti-inflammatory, and antipyretic effects and is used in the treatment of numerous conditions [1]. However, diclofenac use has been associated with significant toxicity, particularly in the liver and kidneys, especially when overdosed [2, 3]. These adverse effects are often associated with oxidative stress and mitochondrial dysfunction [2, 4]. Diclofenac induces oxidative damage by producing reactive oxygen species (ROS) [5, 6]. ROS cause lipid peroxidation and damage cellular macromolecules, thereby contributing to organ injury [5, 6]. Moreover, ROS, particularly mitochondrial hydrogen peroxide, plays key roles in diclofenac-induced liver toxicity by causing damage and impairing mitochondrial function [2]. This leads to apoptosis and exacerbates oxidative stress. Additionally, recent research has indicated that diclofenac disrupts autophagic flux, which is a crucial process for maintaining cellular homeostasis and mitochondrial integrity [7]. Autophagy disruption hinders the removal of damaged mitochondria, resulting in increased oxidative stress and hepatotoxicity [7]. Recently, based on their anti-inflammatory and glucose- and monocarboxylate transporter inhibitory properties [8, 9], nonsteroidal anti-inflammatory drugs were proposed for treating various types of cancers, either as monotherapy or in combination with established or experimental anti-cancer drugs [10]. The underlying mechanisms have been suggested to involve the prevention of tumor-promoting inflammatory processes in the tumor microenvironment and modulation of tumorigenic metabolic remodeling by inhibiting the said transporters [8, 9].

Olaparib, a poly (ADP ribose) polymerase (PARP) inhibitor primarily used as a cancer therapeutic compound due to its selective toxicity toward breast cancer1 (BRCA1) and BRCA2-mutated cancer cells [11]. Additionally, it has shown potential for reducing oxidative stress and inflammation in non-oncological models [12, 13]. Furthermore, it enhances autophagy and mitophagy, which are essential processes for maintaining mitochondrial integrity, by removing damaged mitochondria [14]. Furthermore, a low dose of olaparib improves cardiac function under septic conditions by reducing ferroptosis through increased mitophagy flux [15]. By promoting mitophagy, olaparib helps maintain mitochondrial quality, which is vital for cell survival under oxidative stress [16]. These findings suggest that olaparib alleviates oxidative stress-related damage in various contexts by promoting mitochondrial health.

A recent study reported that olaparib disrupted oxidative phosphorylation by inhibiting complex I [17], which effect was separate from its PARP inhibitory property. It raises the possibility that olaparib and diclofenac may perturb cellular energy production in cancer cells by impeding both oxidative phosphorylation and glycolysis, respectively. In addition, the metabolic effects of these drugs seem relevant to their cytotoxicity and cytoprotection considering that olaparib has been reported to relieve cellular oxidative stress [18]. Accordingly, this study aimed at further characterizing the role of mitochondrial processes in diclofenac toxicity by using olaparib as a putative mitochondria-protective agent. We examined the effects of olaparib and diclofenac on the cell death process, oxidative stress, and mitochondrial functions in a cell culture model using HepG2 human hepatoma cells. This approach had an additional advantage of providing information about the feasibility of the use of these drugs in combination for oncological applications.

## Materials and methods

### Chemicals

All chemicals were of the highest quality available, and if not stated otherwise were purchased from Sigma-Aldrich (Budapest, Hungary). Diclofenac and 2’,7’-dichlorodihydrofluorescein diacetate (H_2_DCFDA) were purchased from MedChem Express (Princeton, NJ, USA). Olaparib was purchased from Energy Chemicals (Zhoushan, China). Apoptosis-inducing factor (AIF) and glutathione peroxidase 4 (GPx4) monoclonal antibodies were purchased from Santa Cruz Biotechnology (Heidelberg, Germany).

### Cell culture and treatments

HepG2 cells were grown in Minimum Essential Medium supplemented with 10% fetal bovine serum (FBS). AML12 immortal, epithelial mouse ​liver cell line was cultured in high glucose Dulbecco’s Modified Eagle Medium supplemented with 10% FBS. Both cell types were maintained in an incubator at 37 °C with 5% CO_2_ atmosphere. The cells were cultured until 80–90% confluence before splitting, were plated a night before the experiment and were treated with 200 µM diclofenac, 40 µM olaparib, 100 µM NAD^+^, 0.15- or 1-mM N-acetyl cysteine (NAC) and 0.7 or 1 µM rotenone.

### Cell viability analyses

The trypan blue exclusion assay was used to distinguish between live and dead cells. After the treatment, trypan blue solution was immediately added to the cells at a final concentration of 0.4% and bright and blue cells were counted separately under a light microscope using a hemocytometer.

The 3-(4,5-Dimethylthiazol-2-yl)-2,5-diphenyltetrazolium bromide (MTT) assay was used to assess cell viability. HepG2 and AML12 cells were plated at a density of 5 × 10^3^ into 96-well plateson the day before treatment. After the treatment, the cells were incubated with 12 µL MTT for 3 h, and the resulting formazan crystals were solubilized by gently agitating the pellet in 10% sodium dodecylsulphate for 15 min. A microplate reader was used to measure the optical density at 570 nm.

The real-time cytotoxicity analysis (RTCA) xCELLigence system (Agilent, USA) was used to assess cell proliferation and invasive growth of HepG2 cells. The xCELLigence system measures the cell index that reflect cell proliferation and invasive growth by monitoring the real-time impedance of the incubation chamber [19].

### Immunofluorescence analysis

After the treatment, HepG2 cells were fixed with 3% paraformaldehyde for 18 min and then exposed to a permeabilization solution containing Triton X-100 for 15 min at room temperature. The cells were then incubated with the primary antibody overnight at 4 °C, followed by a one-hour incubation with a fluorescent secondary antibody at room temperature. Hoechst staining was used for nuclear staining. Images were captured using a C2 confocal fluorescence microscope (Nikon, Japan).

### Measurement of mitochondrial membrane potential (ΔΨm)

Following incubation in 48-well plates, HepG2 and K562 cells (3 × 10^5^ cells/well) were treated with JC-1 membrane potential sensitive fluorescent probe for 20 min. The cells were rinsed twice with PBS and imaged using a fluorescence microscope (Nikon, Japan). In each well, the fluorescence of aggregated JC-1 (red) and JC-1 monomers (green) was detected at excitation and emission wavelengths of 560/595 nm and 485/535 nm, respectively. Average red and green fluorescence of all cells in the same microscopic field was calculated by the microscope’s software, and their ratio was used to assess ΔΨm. For flow cytometry, the cells were loaded with the JC-1 probe for 20 min, and the mean red and green fluorescence of all cells was determined using flow cytometry (Sony, Japan).

### Assessment of cellular energy metabolism

The mitochondrial oxygen consumption rate (OCR) and extracellular acidification rate (ECAR) were assessed using a Seahorse XFp analyzer (Agilent, USA). The cells were plated at a density of 10,000 cells/well in 8-well XFp cell culture microplates and treated according to the manufacturer’s protocol. The cells were treated as indicated for 14 h and the OCR and ECAR were recorded every 3 min over a 90-minute period, during which 1 µM oligomycin, 0.125 µM carbonyl cyanide 4-(trifluoromethoxy) phenylhydrazone (FCCP), and 1 µM rotenone/antimycin were added at the indicated time points.

### ROS measurement

To evaluate the intracellular ROS levels, the oxidation of non-fluorescent H_2_DCFDA was used. Briefly, 2 × 10^4^ cells were treated as indicated, the probe was then added to the culture medium for 20 min at 37 °C, and the cells were live imaged under a fluorescent microscope.

### Fenton reaction

The Fenton reaction was performed by adding 100 mM H_2_O_2_ to 100 mM of FeSO_4_ solution in pH 4.5 acetate buffer containing 5 mM sodium salicylate in the presence or absence of 100 µM olaparib or 5 mM NAC. Photometry measurements were performed on a Perkin-Elmer FL6500 spectrofluorometer (Per-Form Hungaria, Budapest, Hungary) using 1 mL quartz cuvettes at room temperature at the wavelength of 510 nm in photometry setup.

### Statistical analysis

Data is presented as mean ± standard deviation (SD) and were evaluated using GraphPad Prism version 10. One-way analysis of variance was performed to assess statistically significant differences among the experimental groups, followed by Tukey’s post-hoc test. Differences between groups were considered statistically significant at *p* < 0.05.

## Results

### Olaparib reduced diclofenac-toxicity and enhanced invasive growth of HepG2 cells

**Fig. 1:**
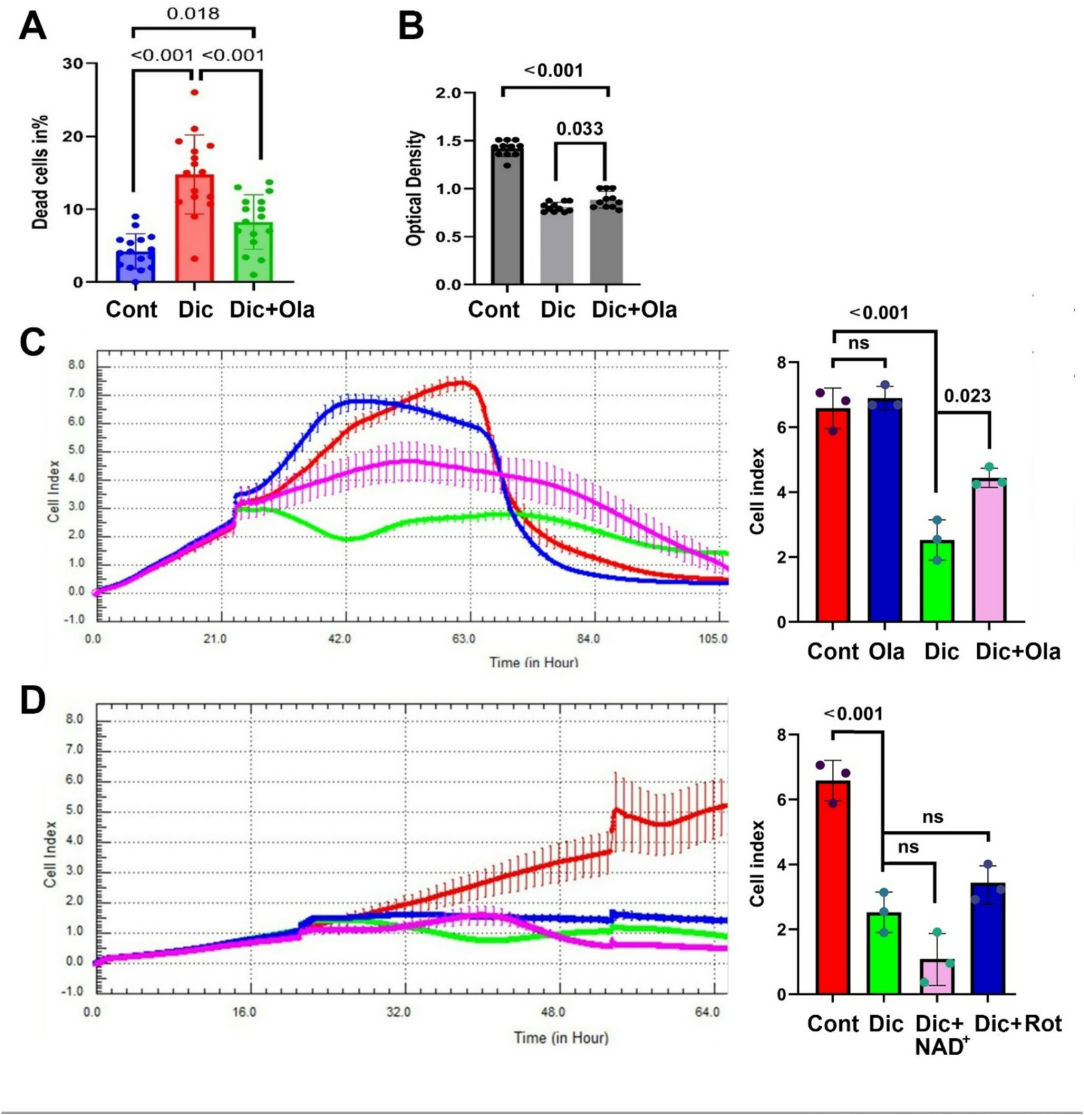
Olaparib diminished diclofenac-induced cell death. **A** and **B** Viabilities of HepG2 presented as ratio of dead cells (**A**) and of AML12 (**B**) cells were assessed by trypan blue exclusion (**A**) and MTT (**B**) assays after treating the cells with different combinations of diclofenac (Dic, 200 µM) and olaparib (Ola, 40 µM) for 24 h. Data is given as mean ± SD of three independent experiments running in at least three parallels (*n* = 9–16). **C** and **D** RTCA assay was used to assess the effect of the drugs on the viability and invasive growth of HepG2 cells. The cells were treated with diclofenac (Dic, 200 µM), olaparib (Ola, 40 µM), NAD^+^ (100 µM) and rotenone (Rot, 1 µM) in the indicated combination for up to 120 h. Cell index calculated from the curves that reflect invasive growth is presented as mean ± SD of three independent experiments running in at least three parallels (*n* = 9–12). Calculated p values are indicated above the bars. Differences between the groups were considered significant at *p* < 0.05. ns, non-significant difference

The effects of olaparib, diclofenac, and their combination on the viability of HepG2 cells was determined by trypan blue stain after treating the cells with the indicated combination of the drugs for 24 h (Fig. 1A). Olaparib alone did not affect HepG2 cell viabilities (not shown here), however, it significantly reduced diclofenac toxicity (Fig. 1A). We used another cell line derived from hepatocytes of normal liver of a 3-month-old male CD1 mouse (AML12) to demonstrate that diclofenac’s toxic and Olaparib’s protective effects are not restricted to cancerous or human hepatocytes only (Fig. 1B). To further characterize the effect of drugs, proliferation and invasive growth of the cells were evaluated using the RTCA assay. HepG2 cells were seeded on E-plates one day before the application of the drugs to allow adherence of the cells to the plates. The cells were incubated in the presence of drugs for up to 120 h, during which time the cell indices reflecting proliferation and invasive growth were monitored. Instead of NAD^+^, its precursor, nicotinamide riboside was added to the medium at the concentration indicated for NAD^+^. Even during this long exposure, olaparib alone did not affect proliferation and invasive growth of the HepG2 cells, while protected them from diclofenac toxicity (Fig. 1C). Since the inhibition of PARP conserves NAD^+^ [20], and olaparib was reported to inhibit complex I of the mitochondrial respiratory chain [17], we tested the effect of NAD^+^ and rotenone, a complex I inhibitor, on diclofenac’s cytotoxicity. Although NAD^+^ tended to antagonize the detrimental effect of diclofenac by 40 h of incubation, on the longer run it exaggerated it, while rotenone demonstrated an overall protective tendency. However, these changes did not reach the level of statistical significance (Fig. 1D).

### Olaparib ameliorated mitochondrial depolarization in diclofenac-induced toxicity

**Fig. 2:**
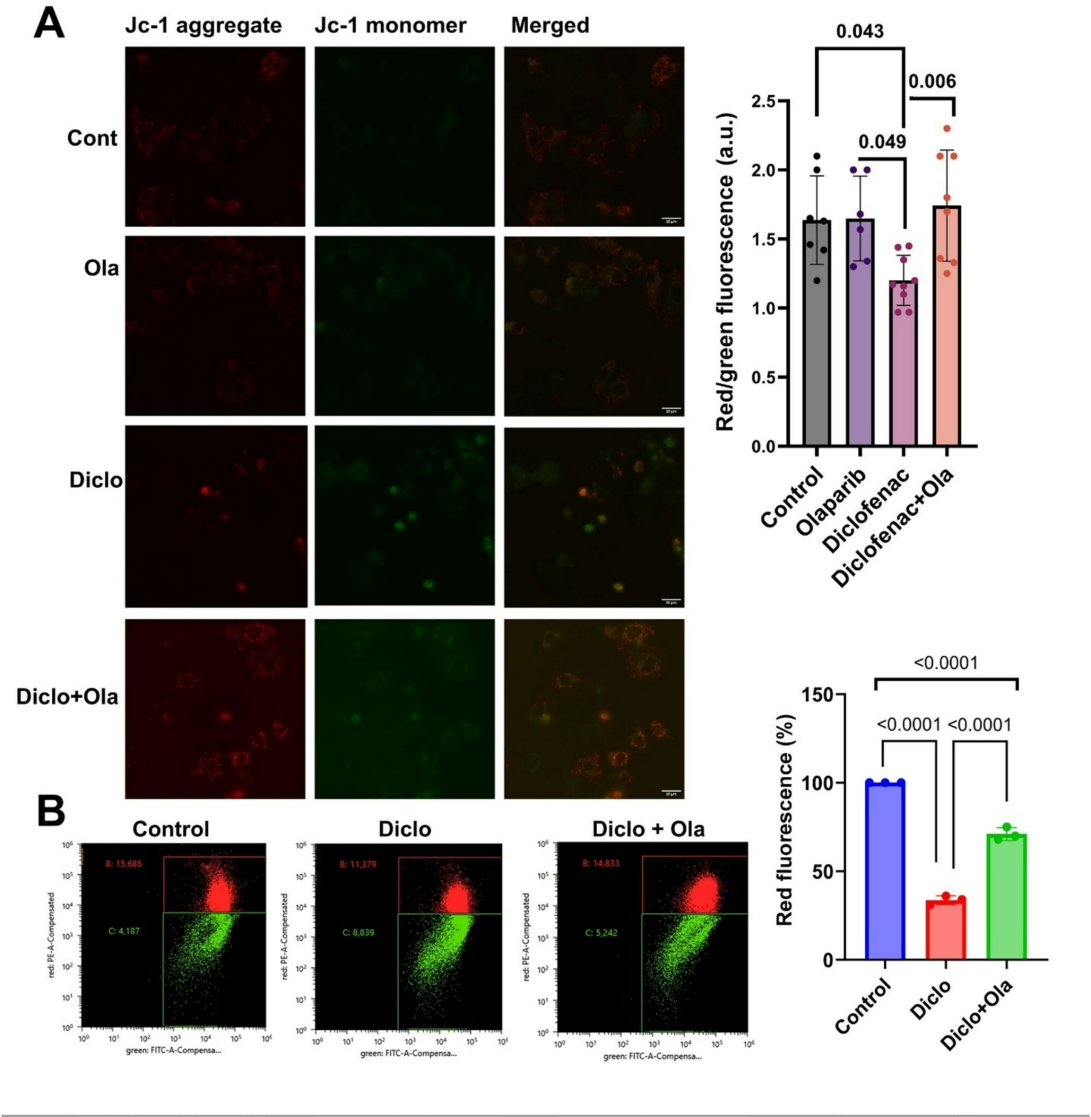
Olaparib ameliorated diclofenac induced mitochondrial depolarization. **A** HepG2 cells were treated with diclofenac (Diclo, 200 µM) and olaparib (Ola, 40 µM) in the indicated combination for 20 h, and live-imaged by fluorescent microscopy after loading with the membrane potential dependent fluorescent dye, JC-1. The results are presented as representative microscopy images of identical fields in the red (aggregated JC-1 indicating polarized mitochondria) and green (monomeric JC-1 indicating depolarized mitochondria) channels. Scale bars in the lower right corner of the merged images represent 25 μm. The accompanying bar diagram presents the average ratio of red to green fluorescence intensity in arbitrary units (a.u.), mean ± SD of three separate experiments running in 2–3 parallels (*n* = 6–9). (B) K562 cells were treated with diclofenac (Diclo, 200 µM) and olaparib (Ola, 40 µM) in the indicated combination for 22 h, stained with JC-1 dye, and red and green fluorescence intensities were determined using flow cytometry. Data are presented as red fluorescence in the % of total fluorescence, mean ± SD of three independent experiments (*n* = 3). Calculated p values are indicated above the bars. Differences between the groups were considered significant at *p* < 0.05

The effects of the drugs on mitochondrial depolarization were measured using JC-1, a membrane potential-sensitive fluorescent dye. After treating HepG2 or K562 cells with different combinations of 200 µM diclofenac and 40 µM olaparib for 20 h, we assessed the mean JC-1 fluorescent intensities in the red and green channels of a fluorescence microscope. We used the red/green intensity ratio to demonstrate mitochondrial depolarization (Fig. 2A). Flow cytometric analysis was also performed on non-adherent K562 leukemia cells (Fig. 2B). The ΔΨm was determined by evaluating the mean intensity of red and green fluorescence and expressing red fluorescence as the percentage of all (red+green) fluorescence (Fig. 2B). Diclofenac treatment significantly decreased the ΔΨm in both cell lines (Fig. 2). However, when olaparib was used in combination with diclofenac, the ΔΨm aggregate level significantly increased compared with ΔΨm levels with diclofenac alone (Fig. 2). This finding suggests that olaparib effectively counteracted the diclofenac-induced reduction in ΔΨm.

### Olaparib reduced ROS production induced by diclofenac

**Fig. 3:**
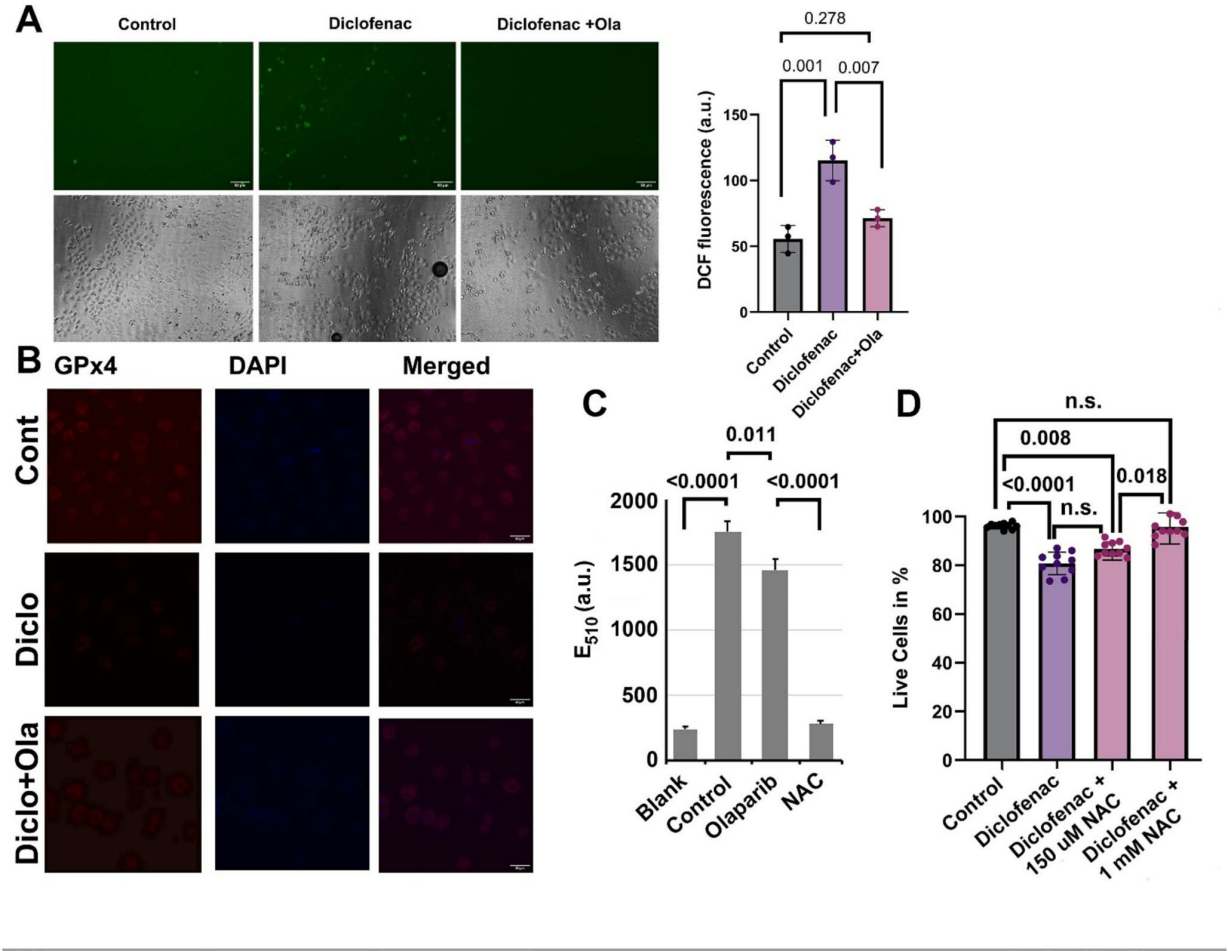
Olaparib reduced diclofenac-induced ROS formation. **A** HepG2 cells were treated with diclofenac (Diclo, 200 µM), olaparib (Ola, 40 µM), or their combination for 24 h, followed by loading the cells with the ROS-sensitive H_2_DCFDA. Fluorescence of 2’,7’-dichlorofluorescein (DCF) resulting from the oxidation of non-fluorescent H_2_DCFDA was assessed using confocal fluorescence microscopy. Data is presented as representative images and as a bar diagram of the DCF fluorescence intensities normalized to a standard and expressed in arbitrary units (a.u.), mean ± SD of three independent experiments (*n* = 3). Bright-field images of the same microscopic field are presented underneath the fluorescent images to indicate cell positions. Scale bars in the lower right corner of the fluorescence images represent 50 μm. **B** Following the same treatment as in (**A**), the cells were fixed, permeabilized, blocked and incubated with anti-GPX4 primary- and anti-mouse secondary antibodies with appropriate wash between steps. Hoechst33342 was used for nuclear counterstaining. Representative immunofluorescence images from three independent experiments (*n* = 3) are shown. Scale bars in the lower right corner of the merged images represent 30 μm. **C** OH^−^ radicals formed in the cell-free Fenton reaction system were measured by photometry. Spontaneous oxidation of salicylic acid was assessed in the absence of H_2_O_2_ (Blank), and the reaction was performed in the absence (Control) and presence of 1 mM Olaparib or 1 mM NAC. Absorbance at 510 nm (E_510_), mean ± SD of three independent experiments (*n* = 3) is presented as a bar diagram. **D** The viability of HepG2 cells was assessed using the trypan blue assay after treating the cells with different combinations of diclofenac (200 µM) and NAC (150 µM or 1 mM) for 24 h. Data is presented as mean ± SD from three independent experiments running in at least three parallels (*n* = 10). Calculated p values are indicated above the bars. Differences between the groups were considered significant at *p* < 0.05. ns, non-significant difference

Massive oxidative stress can lead to cellular damage, and the oxidative processes linked to this damage have been recognized as crucial mechanisms underlying diclofenac-induced toxicity [9]. To explore the role of olaparib in mitigating oxidative stress during diclofenac-induced toxicity, intracellular ROS levels were evaluated. ROS levels were assessed after 24 h of treatment by loading cells with the reduced, non-fluorescent form of the cell-penetrating redox dye, DFCDA. The dye is retained inside the cells after esterases remove acetates and becomes fluorescent upon oxidation by intracellular ROS. Diclofenac induced a notable increase in ROS production compared to the control group, which was counteracted by olaparib (Fig. 3A). One of the major non-enzymatic antioxidant defense mechanisms is the glutathione peroxidase system [21]. We assessed the activity of the glutathione system by GPx4 protein immunofluorescence. We used Hoechst33342 counterstaining to visualize nuclei (Fig. 3B). Diclofenac decreased GPx4 fluorescence that was significantly attenuated by the simultaneous application of olaparib (Fig. 3B). We compared the antioxidant effects of olaparib with those of NAC, a potent free-radical scavenger [22], in a cell-free system. Although olaparib demonstrated significant free-radical scavenging properties in the cell-free Fenton reaction at a concentration of 1 mM, it was much inferior to the effect of equimolar NAC, which eradicated OH^−^ radical formation in this system (Fig. 3C). On the other hand, NAC although showed a tendency of ameliorating the diclofenac induced cell death, at the concentration of 150 µM, it failed to protect significantly the HepG2 cells from diclofenac’s toxicity (Fig. 3D). However, at a concentration of 1 mM, it fully protected the cells (Fig. 3D), indicating that excessive ROS production was involved in the cytotoxicity of diclofenac [23], and thus the reduction in ROS production contributed to the protective effect of olaparib.

### Olaparib mitigated AIF-mediated apoptosis induced by diclofenac

**Fig. 4:**
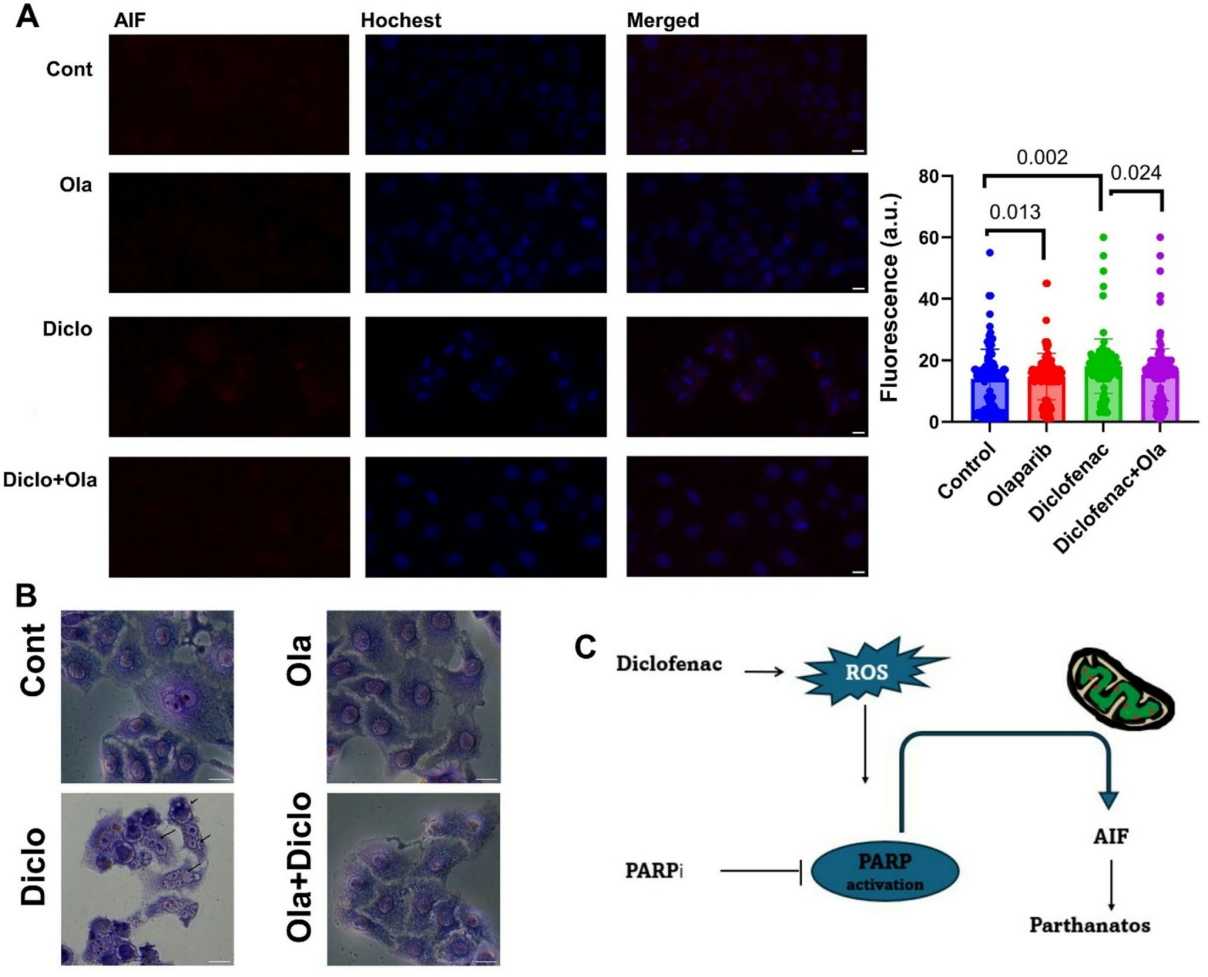
Olaparib diminished diclofenac-induced apoptosis. **A** Following a 20-hour treatment of HepG2 cells with diclofenac (Diclo, 200 µM), olaparib (Ola, 40 µM), or their combination, the cells were fixed, permeabilized, blocked and incubated with anti-AIF monoclonal antibody and anti-mouse secondary antibody with appropriate washings between the steps. Hoechst33342 was used for nuclear staining, and the cells were visualized under a confocal microscope. The results are presented as representative images and as a bar diagram of the fluorescence intensities normalized to a standard and expressed in arbitrary units (a.u.), mean ± SD of three independent experiments (*n* = 3). Scale bars in the lower right corner of the merged images represent 15 μm. Calculated p values are indicated above the bars. Differences between the groups were considered significant at *p* < 0.05. **B** The cells were treated as in (A) for 24 h. Subsequently, they were stained with 3% Giemsa solution for 30 min and were imaged using an inverted microscope. Results are presented as representative images from three independent experiments (*n* = 3). Arrows indicate nuclear fragmentation and chromatin condensation. **C** Schematic illustration of the interactions between ROS, PARP inhibition, and nuclear fragmentation. Pointed arrows indicate activation, whereas flat-headed lines indicate inhibition

Apoptosis-inducing factors are mitochondrial proteins activated or sensitized by PAR polymers. They promote the caspase-independent cell death pathway known as parthanatos [24]. In rats, oxidative stress contributes to diclofenac-induced liver damage, possibly through DNA fragmentation [25]. AIF protein expression was evaluated by immunofluorescence using confocal microscopy. Diclofenac increased the nuclear translocation of AIF, which was diminished by the PARP inhibitor, olaparib (Fig. 4A). To demonstrate apoptosis, HepG2 cells were stained with Giemsa dye to visualize nuclear fragmentation and chromatin condensation. These results demonstrated that diclofenac-induced apoptosis was prevented by olaparib treatment (Fig. 4B). These results suggested that diclofenac-induced ROS production activated PARP. This caused AIF to be released from the area between the mitochondrial intermembrane space. AIF then translocated to the nucleus and initiates parthanatos. All these processes were prevented by olaparib, which inhibited excess PARP activation (Fig. 4C).

### Olaparib decreased the oxidative phosphorylation-inducing effect of diclofenac

**Fig. 5:**
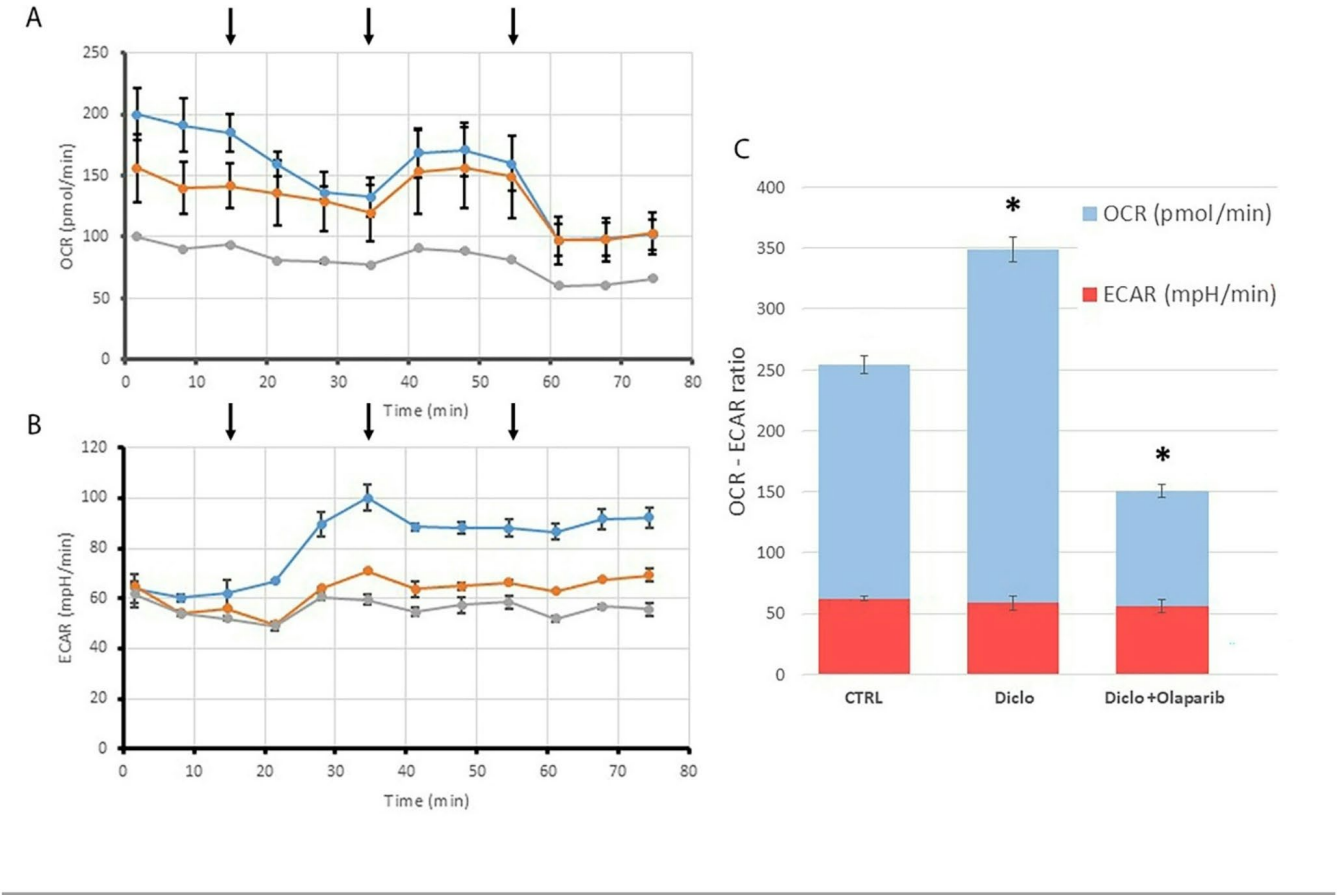
Olaparib *attenuated the oxidative phosphorylation-inducing effect of diclofenac* in HepG2 cells. **A** Original plot generated by the Seahorse cellular respirometer from recordings of OCR, after treating the cells with a combination of diclofenac (Diclo, 200 µM) and olaparib (Ola, 40 µM) for 13.5 h. During each recording, oligomycin, FCCP and rotenone plus antimycin A were added at 15, 35, and 55 min of the run (at the arrows). **B** Original plot of ECAR from the same recordings as for the OCR. (C) OCR/ECAR ratio was calculated from the raw data according to Desousa et al. [26]. All data is presented as mean ± SD, based on three independent experiments (*n* = 3). Asterix indicates a significant difference from the control, *p* < 0.05

To study the effects of diclofenac and olaparib on energy metabolism in HepG2 cells, we used the Seahorse cellular respirometer. HepG2 cells were treated with the indicated combinations of drugs for 13.5 h, and OCR, reflecting oxidative phosphorylation and ECAR, reflecting glycolysis, were measured. The F_o_F_1_ ATPase inhibitor oligomycin, the uncoupler FCCP and the respiratory inhibitors rotenone and antimycin A were added at 15, 35, and 55 min of the run, respectively. The original OCR and ECAR data is shown in Fig. 5A and B, respectively. The OCR/ECAR values (Fig. 5C) were calculated according to Desousa et al. [26] and were used to assess the effects of diclofenac and olaparib on these two ATP-producing pathways. As shown in the figure, neither diclofenac nor the combination of diclofenac and olaparib changed ECAR significantly, whereas olaparib clearly reduced diclofenac-induced OCR (Fig. 5C).

### Oxidative phosphorylation inhibition by rotenone enhanced ΔΨm and cell viability in diclofenac-induced cell toxicity

**Fig. 6:**
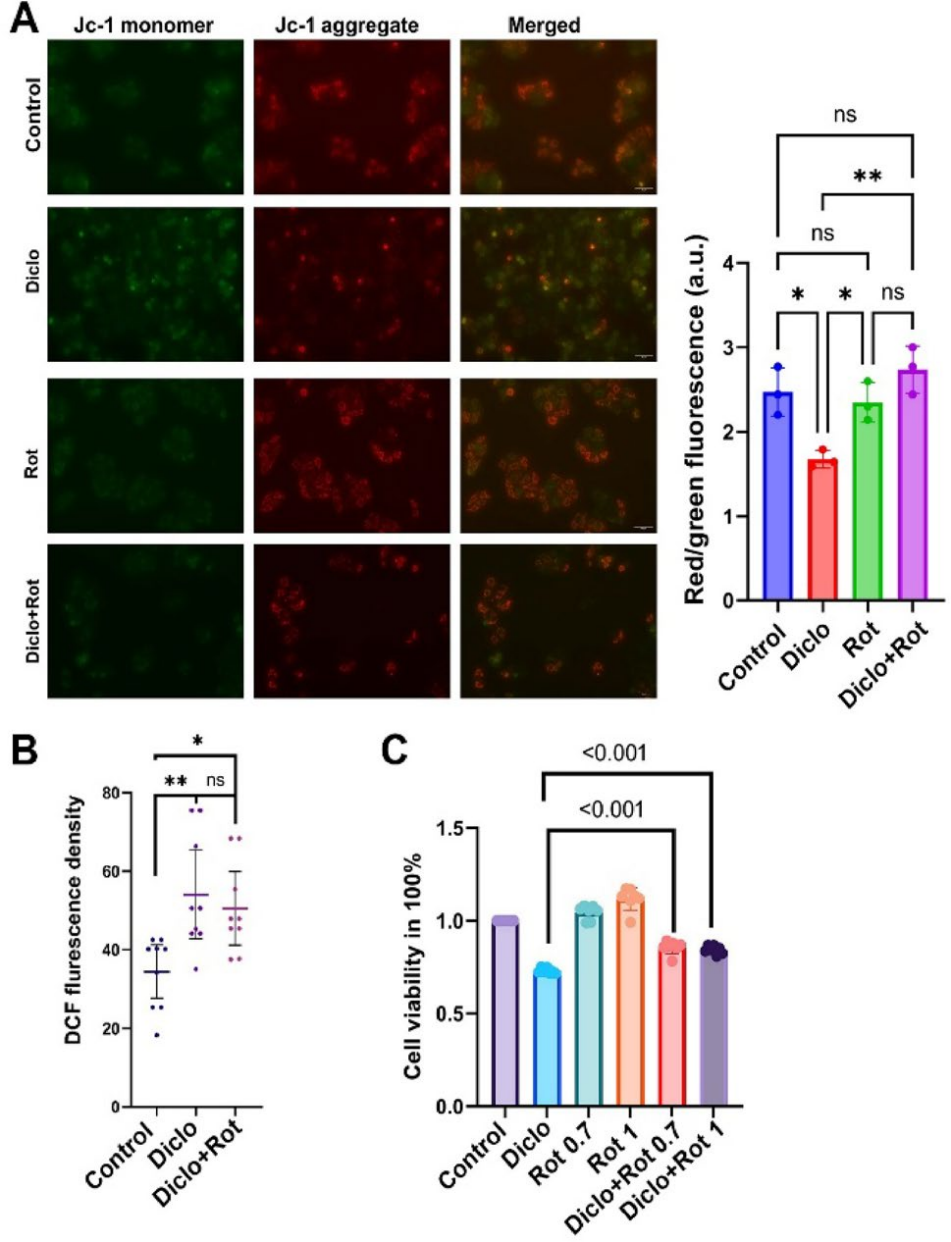
Rotenone enhanced ΔΨm and cell survival in diclofenac-induced toxicity. **A** HepG2 cells were treated with diclofenac (Diclo, 200 M µM) and rotenone (Rot, 1 µM) for 14 h and then live-imaged by fluorescent microscopy after loading them with the membrane potential dependent fluorescent dye, JC-1. The results are presented as representative microscopic images of identical fields in the red and green channels. Scale bars in the lower right corner of the merged images represent 20 μm. The bar diagram presents the average ratio of red to green fluorescence intensity in arbitrary units (a.u.), mean ± SD of three independent experiments (*n* = 3). **B** and **C** HepG2 cells were treated with diclofenac (Diclo, 200 µM) and rotenone (Rot, 0.7 or 1 µM) for 24 h before measuring ROS formation by DCF fluorescence (**B**) and cell viability using the MTT assay (**C**). DCF fluorescence and viability are presented as mean ± SD from seven separate experiments (*n* = 7). Calculated p values are indicated above the bars. Differences between the groups were considered significant at *p* < 0.05. ns, non-significant difference; *, significant difference *p* < 0.05; **, significant difference *p* < 0.01

We demonstrated that rotenone tended to protect HepG2 cells against diclofenac toxicity, however, this effect did not reach the level of statistical significance (Fig. 1D). However, we intended to study how rotenone interacts with diclofenac in more detail because of the reported complex I inhibitory property of olaparib [17]. For determining the effects of rotenone on diclofenac-induced mitochondrial depolarization, we treated HepG2 cells with 200 µM diclofenac and 1 µM rotenone for 14 h before staining the cells with JC-1 and imaging them under a fluorescent microscope. We found that the inhibition of complex I by rotenone reduced diclofenac-induced mitochondrial depolarization (Fig. 6A), similar to olaparib (Fig. 2A). In addition, we determined ROS production and viability of the HepG2 cells after a 24 h exposure to diclofenac and different concentrations of rotenone using the DCF fluorescence and the MTT assays, respectively. Diclofenac induced an approximately 60% increase in ROS production that was not affected significantly by rotenone (Fig. 6B). On the other hand, rotenone alone, at the concentrations of 0.7 and 1 µM did not affect viability of the HepG2 cells significantly, however, it significantly counteracted the cytotoxicity of diclofenac at both concentrations (Fig. 6C).

## Discussion

One of the most notable adverse side effects of chronic diclofenac treatment is hepatotoxicity [1]. The mechanism has been reported to involve ROS production, and mitochondrial damage [27], which are basic cellular mechanisms that do not require interactions among the different cell types of the liver. Accordingly, to investigate the cytotoxicity of diclofenac, we used HepG2 hepatocellular carcinoma cells, which retain various characteristics of hepatocytes [28]. Even though HepG2 is an immortalized cell line and therefore possesses several cancer-specific characteristics, it is still suitable for studying the mechanisms of liver toxicity [29]. In cell culture studies, drugs are often used at supratherapeutic concentrations to induce adverse effects within a short period. Most studies have applied diclofenac at concentrations higher than its IC_50_ of 175 µM [30], even at concentrations as high as 500 µM [8]. We used it at a concentration of 200 µM, which is in agreement with previous studies [2, 9]. At this concentration, diclofenac reduced the viability and invasive growth of HepG2 cells, which were ameliorated by olaparib (Fig. 1A and C). We also demonstrated that this effect of olaparib is not restricted to cancerous or human cells since we had similar results with AML12 immortalized normal mouse hepatocytes (Fig. 1B). These results are consistent with previous studies reporting protective effects of olaparib in hypertension-induced oxidative stress and doxorubicin-induced toxicity [12, 13].

ROS accumulation is crucial in diclofenac-induced liver injury [9, 31]. It disrupts autophagic flux due to ROS buildup and lysosomal dysfunction, which compromises mitochondrial integrity and exacerbates the cellular ROS load [9]. Elevated ROS levels activate PARP-1 via oxidative formation of single-strand DNA damage [24, 25, 32]. Activated PARP-1 uses NAD^+^ as a substrate and ADP-ribosylates its targets thereby initiating DNA repair. However, excessive PARP activation can lead to the depletion of NAD^+^ and ATP, which may cause necrotic death [33]. Therefore, inhibition of PARP by olaparib may preserve NAD^+^ pools [20], which mechanism may contribute to olaparib’s ameliorating effect on diclofenac toxicity. Furthermore, olaparib was reported to inhibit complex I [17] that reduces the rate of NAD^+^ regeneration from NADH and ATP production and decreases free-radical formation [34]. We found that NAD^+^ tended to reduce then to increase diclofenac toxicity after 40 h of incubation (Fig. 1D), but these effects were not significant. However, complex I inhibition by rotenone significantly diminished the ΔΨm loss (Fig. 6A) while it did not affect ROS production (Fig. 6B) induced by diclofenac. We demonstrated that mild inhibition of complex I achieved by treating the cells for 24 h with up to 1 µM rotenone did not affect their viability significantly while it protected them against diclofenac toxicity (Fig. 6C). However, this protective effect of complex I inhibition remained a tendency that did not reach the level of statistical significance at longer exposures (Fig. 1D). Taken together, these results indicate that the protective effect of olaparib in diclofenac toxicity was unlikely to involve complex I inhibition.

Under stress situations, adverse cellular signaling leads to mitochondrial damage, eventually leading to parthanatos, a unique type of programmed cell death characterized by the overactivation of PARP and nuclear translocation of AIF [35]. This leads to extensive nuclear fragmentation and chromatin condensation, ultimately resulting in cell death [36, 37]. In line with this phenomenon, we found that olaparib, which had moderate free-radical scavenging properties in a cell-free system as compared with equimolar NAC, diminished diclofenac-induced ROS production in HepG2 cells at a concentration of 40 µM with an efficacy similar to that of 1 mM NAC (Fig. 3). This difference in the antioxidant efficacy of olaparib in live cells compared to a cell-free system indicates that the antioxidant property of olaparib is unlikely to result from direct scavenging activity. Instead, it is the result of affecting protective cellular mechanisms, most notably, mitochondrial functions [38, 39]. In addition, olaparib reduced diclofenac-induced nuclear translocation of AIF and AIF-mediated nuclear fragmentation (Fig. 4). We did not study the mechanism, by which the PARP inhibitor olaparib exerts these effects, because the cytoprotective mechanisms of PARP inhibition have been extensively characterized previously [39, 40].

Diclofenac triggers the formation of mitochondrial permeability transition pore (mPTP) in isolated mitochondria and rat hepatocytes [41, 42]. Mitochondrial membrane depolarization is associated with mPTP [43]. Both mPTP and mitochondrial membrane potential dissipation induce the release of pro-apoptotic intermembrane proteins [44]. In contrast, olaparib, by modulating intracellular signaling [38], protects the mitochondria and maintains ΔΨm under stress conditions, such as hypoxia and oxidative stress [12, 39]. It effectively mitigates the hypoxia-induced collapse of ΔΨm in retinal cells and prevents depolarization of the mitochondrial membrane caused by ROS in peripheral white mononuclear cells, thereby restoring ΔΨm [45]. We found that ΔΨm levels were significantly increased by the combination of diclofenac and olaparib compared to diclofenac alone (Fig. 2) suggesting that olaparib effectively counteracted diclofenac-induced mitochondrial depolarization.

Remodeling energy metabolism of the cells represents an additional mechanism underlaying diclofenac’s cytotoxicity. Recently diclofenac has been suggested for cancer therapy [5] owing to its anti-inflammatory properties that prevent tumor-promoting inflammatory processes in the tumor microenvironment and its metabolic remodeling effect [6, 7]. The latter involves the induction of mitochondrial ATP production, because cancer cells predominantly depend on aerobic glycolysis for ATP generation [46]. Diclofenac was reported to decrease glucose transporter 1 expression and hexokinase activity, consequently diminishing glucose uptake and glycolytic activity in triple-negative breast cancer cells [47]. In addition, diclofenac inhibits monocarboxylate transporter 1 (MCT1) and lactate dehydrogenase A (LDHA) activities thereby obstructing the conversion of pyruvate to lactate, a crucial step in glycolysis [48]. Consequently, diclofenac lowers lactate production, disrupts the hypoxic environment within the tumor, and induces apoptosis. A reduction in ATP levels induces oxidative stress in mitochondria and activates AMP-activated protein kinase signaling, which further promotes apoptosis. The alleviation of diclofenac-induced cytotoxicity largely relies on LDHA inhibition, as cells with depleted LDHA protein expression show resistance to diclofenac-induced cell death [30, 48]. In agreement with this phenomenon, we found that diclofenac increased the OCR but did not affect the ECAR (Fig. 5C). The suppressive effect of diclofenac on MCT1 [48] may account for the latter finding. We found that olaparib counteracted the diclofenac-induced increase in OCR but did not affect ECAR (Fig. 5C). Additionally, the data demonstrating that the complex I inhibitor, rotenone, had protective effects similar to those of olaparib (Fig. 6) indicated the importance of the induction of oxidative phosphorylation in diclofenac-induced toxicity. However, these results also suggest that olaparib may interfere with the metabolic reprogramming effect of diclofenac; therefore, the feasibility of combining diclofenac with olaparib for oncological applications must be further investigated.

In conclusion, we established, to the best of our knowledge for the first time, that the PARP inhibitor, olaparib, effectively alleviates diclofenac cytotoxicity, and demonstrated the role of oxidative stress and mitochondrial function in this process. These results also suggest that diclofenac and olaparib may have contrasting effects on the metabolic reprogramming of cancer cells, indicating the need for further studies before considering their combined use for oncological applications.

## Data Availability

The datasets generated during and/or analysed during the current study are available upon request from the corresponding author.

## Abbreviations

AIF: Apoptosis-inducing factor
ΔΨm: Mitochondrial membrane potential
ECAR: Extracellular acidification rate
FBS: Fetal bovine serum
GPx4: Glutathione peroxidase 4
H2DCF-DA: 2’,7’-dichlorodihydrofluorescein diacetate
LDHA: Lactate dehydrogenase A
mPTP: Mitochondrial permeability transition pore
MTT: 3-(4,5-dimethylthiazol-2-yl)-2,5-diphenyltetrazolium bromide
NAC: N-acetyl cysteine
ns: Non-significant differences
OCR: Oxygen consumption rate
PARP: Poly (ADP-ribose) polymerase
ROS: Reactive oxygen species
RTCA: Real-time cell analysis
SD: Standard deviation
